# Graphene Oxide-Magnetic Nanoparticles Loaded Polystyrene-Polydopamine Electrospun Nanofibers Based Nanocomposites for Immunosensing Application of C-Reactive Protein

**DOI:** 10.3390/bios12121175

**Published:** 2022-12-16

**Authors:** Simge Ketmen, Simge Er Zeybekler, Sultan Sacide Gelen, Dilek Odaci

**Affiliations:** Department of Biochemistry, Faculty of Science, Ege University, Bornova, Izmir 35100, Turkey

**Keywords:** nanobiotechnology, nanotechnology, graphene oxide, magnetic nanoparticles, polydopamine, polystyrene, electrospun nanofiber

## Abstract

The large surface area/volume ratio and controllable surface conformation of electrospun nanofibers (ENFs) make them highly attractive in applications where a large surface area is desired, such as sensors and affinity membranes. In this study, nanocomposite-based ENFs were produced and immobilization of Anti-CRP was carried out for the non-invasive detection of C-reactive protein (CRP). Initially, the synthesis of graphene oxide (GO) was carried out and it was modified with magnetic nanoparticles (MNP, Fe_3_O_4_) and polydopamine (PDA). Catechol-containing and quinone-containing functional groups were created on the nanocomposite surface for the immobilization of Anti-CRP. Polystyrene (PS) solution was mixed with rGO-MNP-PDA nanocomposite and PS/rGO-MNP-PDA ENFs were produced with bead-free, smooth, and uniform. The surface of the screen-printed carbon electrode (SPCE) was covered with PS/rGO-MNP-PDA ENFs by using the electrospinning technique under the determined optimum conditions. Next, Anti-CRP immobilization was carried out and the biofunctional surface was created on the PS/rGO-MNP-PDA ENFs coated SPCE. Moreover, PS/rGO-PDA/Anti-CRP and PS/MNP-PDA/Anti-CRP immunosensors were also prepared and the effect of each component in the nanocomposite-based electrospun nanofiber (MNP, rGO) on the sensor response was investigated. The analytic performance of the developed PS/rGO-MNP-PDA/Anti-CRP, PS/rGO-PDA/Anti-CRP, and PS/MNP-PDA/Anti-CRP immunosensors were examined by performing electrochemical measurements in the presence of CRP. The linear detection range of PS/rGO-MNP-PDA/Anti-CRP immunosensor was found to be from 0.5 to 60 ng/mL and the limit of detection (LOD) was calculated as 0.33 ng/mL for CRP. The PS/rGO-MNP-PDA/Anti-CRP immunosensor also exhibited good repeatability with a low coefficient of variation.

## 1. Introduction

C-reactive protein (CRP) is an acute-phase protein that is synthesized by the liver due to infection and is an important component of the immune system [1]. Today, electrochemical immunosensor systems have become the devices needed for clinical applications due to their very fast sensing, fast response times, and small sample requirement [2]. The key to the successful application of electrochemical immunosensors is to design new systems that can convert low limit of detection (LOD) and high sensitivity recognition information into detectable signals [3]. Due to their extraordinary properties, nanomaterials can increase the effective surface area of the electrodes and the electron transfer rate on the modified surface [4]. The usage of nanomaterials such as graphene oxide (GO), graphene quantum dots, carbon nanotubes, and fullerene is becoming increasingly common for modification of SPCE surfaces [5]. All these carbon nanomaterials have their own unique properties. This allows the development of bioanalytical systems with the desired properties.

Electrospun nanofibers (ENFs), which are derived from an electrohydrodynamic process, have superior properties such as large surface area, low diffusion barrier [6], and a controllable surface conformation [7]. ENFs are produced from polymer solutions using the electrospinning technique under a high electric field (kV). ENFs offer an immobilization matrix for the conjugation of biomolecules such as antibodies [8], enzymes [9], and aptamers [10]. Additionally, the functionalization of ENFs with graphene-based nanomaterials provides improved active reaction regions, regioselectivity, and conducting network on the electrode surface for various biological molecules [11]. Thus, immunosensor platforms can be produced with better biosensing performance. Moreover, graphene-based nanomaterials can be modified with various metals, metal oxides, and gold nanoparticles. Thus, good synergy effects can be obtained with increased specific surface area, and improved electron and mass transportation for the development of sensor systems [12,13].

Graphene is a two-dimensional (2D) carbon inexpensive nanomaterial with a single-layer structure [14]. Graphene normally has two degrees of oxidation: GO and reduced graphene oxide (rGO). GO has low conductivity and is water soluble, while rGO has good conductivity and poor water solubility [15]. The presence of oxygen-rich functional groups causes a decrease in electrical conductivity. Therefore, GO is usually reduced for sensor applications. In this way, π-conjugation in the graphene sheets restores the conductivity of the graphene [16]. However, the aggregation of rGO in the polymer solution can occur due to intermolecular π–π interactions and van der Waals forces when conventional nanocomposite synthesis methods are used (solvent processing, in situ polymerization, etc.). Electrospinning offers an easy and effective way of integrating graphene-based nanomaterials into the structures of polymers [12].

Dopamine (DA) is a neurotransmitter with catechol and amine functional groups that can be polymerized under alkaline conditions (pH = 8.5) [17]. Polydopamine (PDA) coating can be obtained on solid substrates with catechol-containing and quinone-containing active groups [18]. PDA coating has two important advantages. It can be formed on organic and inorganic surfaces through covalent and non-covalent interactions. Moreover, it allows immobilization of thiol- or amine-containing biomacromolecules on the PDA [19]. There are various studies about the immobilization of peptides [19], DNA [20], enzymes [21], and antibodies [22] on PDA-modified platforms. These studies showed that the PDA can be used not only for immobilization platforms but can also be used as a cross-linker agent [23,24]. Thanks to this superior feature, PDA provides an advantage in the development of new immunosensor systems.

Iron oxide (Fe_3_O_4_) magnetic nanoparticles (MNP), incorporating a semi-metallic metal oxide with an inverted spinel structure, have excellent adsorption capacity, high electrocatalytic properties, magnetic and natural electrical conductivity [25]. The placement of Fe_3_O_4_ nanocomposites on the electrode surface results in increased sensitivity, and most crucially, increased response to noise ratio [26,27]. In addition, Fe_3_O_4_-based electrochemical sensors have remarkable features such as high sensitivity, low cost, and fast response time [28].

In this study, besides the development of a new immunosensor for non-invasive detection of CRP from artificial human saliva, the effect of each nanomaterial on the sensor response in the PS/rGO-MNP-PDA nanofiber was also investigated. PS/rGO-MNP-PDA, nanofibers were produced as an immobilization matrix using electrospinning technique for the Anti-CRP immobilization. Saliva allows the development of point of care (POC) devices due to the non-invasiveness, speed, and stress-freeness of the sampling procedure for the patient [29]. A good peak separation, excellent catalytic activity, and large surface area can be obtained by combining MNP and carbonaceous materials such as rGO. Additionally, this combination prevents self-aggregation of MNP and leads to enhanced electrical conductivity [26]. PS is not useful as an immobilization matrix for biological molecules via covalent bonds due to its highly hydrophobic nature [30]. For this reason, rGO-MNP-PDA nanocomposite was synthesized and combined with PS solution before the electrospinning process. Thus, the hydrophilic surface and functional groups were created on the nanofibers via catechol-containing and quinone-containing species of PDA. The surface of the working electrode was covered with PS/rGO-MNP-PDA nanofibers using electrospinning technique under the determined optimum conditions and Anti-CRP immobilization was carried out directly on the PS/rGO-MNP-PDA nanofibers modified SPCE. For the characterization of PS/rGO-MNP-PDA nanofibers after each modification step, Scanning Electron Microscopy-Energy Dispersion X-ray Spectrometer (SEM-EDS), Fourier transform infrared spectroscopy (FTIR), and contact angle measurements were performed. The characterization of PS/rGO-MNP-PDA/Anti-CRP immunosensor was examined with electrochemical measurements [differential pulse voltammetry (DPV), cyclic voltammetry (CV) and electrochemical impedance spectroscopy (EIS) techniques] in the presence of CRP. Moreover, PS/rGO-PDA/Anti-CRP and PS/MNP-PDA/Anti-CRP were also prepared and the effect of each component in the nanocomposite-based electrospun nanofiber (MNP, rGO) on the sensor response was investigated using the DPV technique in the presence of CRP.

## 2. Material and Methods

### 2.1. Chemicals

C-Reactive Protein Antibody (Anti-CRP, MABF768) was obtained from Merck. Recombinant Human CRP Protein (PRO-335) was purchased from ProSpec. Polystyrene (PS, average Mw~350,000), graphite powder (GR, <20 μm, synthetic), concentrated sulfuric acid (H_2_SO_4_) 95.0–98.0% (w/w), phosphoric acid (H_3_PO_4_) 85% (w/w), KMnO_4_ (99%), H_2_O_2_ solution (30%), FeCl_3_·6H_2_O (97%), FeCl_2_·4H_2_O (99%), dopamine hydrochloride (DA, 98%), urea (99.0–100.5%), bovine serum albumin (BSA, ≥98.0%), insulin (INS), serum amyloid A (SAA), potassium chloride (KCl), sodium chloride (NaCl), potassium thiocyanate (KSCN), potassium phosphate monobasic (KH_2_PO_4_), sodium sulfate decahydrate (Na_2_SO_4_·10H_2_O), ammonium chloride (NH_4_Cl), calcium chloride dihydrate (CaCl_2_·2H_2_O), sodium bicarbonate (NaHCO_3_), potassium hexacyanoferrate (III) (HCF, K_4_[Fe(CN)_6_]), and *N*,*N*-dimethylformamide (DMF) were purchased from Sigma-Aldrich. All chemicals were used without further purification.

Artificial saliva which consists of 0.126 g/L NaCl, 0.964 g/L KCI, 0.189 g/L KSCN, 0.654 g/L KH_2_PO_4_, 0.200 g/L urea, 0.763 g/L Na_2_SO4·10H_2_O, NH_4_Cl 0.178 g/L, 0.228 g/L CaCl_2_·2H_2_O, 0.630 g/L NaHCO_3_ was prepared according to the literature [31].

### 2.2. Instruments

NanoWeb Electrospun 103 (MaviTech) was used for the electrospinning process. New Era Pump System was used as a syringe pump in this process. ATR-FTIR (Perkin Elmer Spectrum Two for the range of 4000 and 600 cm^−1^) was used to investigate GO, rGO-MNP, rGO-MNP-PDA nanocomposites, and PS/rGO-MNP-PDA electrospun nanofibers. The contact angle measurements for the PS/rGO-MNP-PDA electrospun nanofibers were examined with the Attension Theta Goniometer (USA) apparatus. SEM-EDS was used to examine morphological and elemental composition properties of PS/rGO-MNP-PDA ENFs (Thermo Scientific Apreo).

The coefficient of variation (*cv*) value of the nanofiber diameters was determined using Equation (1) to examine how the standard deviation of nanofiber diameters varies with the mean.

(1) $$cv=\frac{\sigma }{µ}$$

where *σ* symbolizes the standard deviation (SD) and μ symbolizes the average diameter of fibers [32].

CV, DPV, and EIS techniques were used for electrochemical measurements using a PalmSens4 potentiostat (Palm Instruments) with SPCE (C110-carbon working electrode (4 mm diameter), reference electrode (Ag), and auxiliary electrode (carbon) (Metrohm).

### 2.3. Synthesis of GO and rGO-MNP

GO was obtained using the improved Hummers method from GR [33]. Briefly, 1.0 g GR, 6.0 g KMnO_4_ were mixed in H_2_SO_4_: H_3_PO_4_ (9:1, v/v) for 12 h at 50 °C. The obtained solution was added to ice (~130 mL frozen distilled water) and 1 mL of 30% (w/v) H_2_O_2_ mixture. Next, the centrifuge process was performed at 6226 rcf (g) for 4 h. The obtained GO was washed with 10% (w/v) HCl solution to remove metal ions, ethanol and left to dry at 60 °C overnight.

The rGO–MNP nanocomposite was obtained using the co-precipitation and ultrasound-assisted method in the presence of GO [8,17]. Firstly, 1.0 mg/mL GO aqueous solution (50 mL) was sonicated in ultrasonic bath (ultrasonic power: 200 W, ultrasonic frequency: 37 kHz) for 30 min. At the same time, 4.0 mmol FeCl_3_·6H_2_O and 2.0 mmol FeCl_2_·4H_2_O were added to 50 mL of an ultra-pure water, then sonicated for 5 min. Then, both solutions were mixed and sonicated again for 5 min. After the sonication process, the nanocomposite solution was placed on a magnetic stirrer and heated to 80 °C. Then, 3 mL ammonia solution (25%, w/v) was added to the nanocomposite solution drop by drop and vigorously stirred (600 rpm) for another 30 min. After this process, the nanocomposite solution was sonicated for 30 min at room temperature again, then rGO-MNP was obtained by precipitation at 14.000 rcf (g), washed with distilled water two times, and left to dry at 60 °C for 2 h.

### 2.4. Preparation of rGO-MNP-PDA, rGO-PDA, and MNP-PDA

200 mg rGO-MNP was added into Tris buffer (0.01 M, pH 8.5), and sonicated (ultrasonic bath (ultrasonic power: 200 W, ultrasonic frequency: 37 kHz) for 30 min. Then, 400 mg DA was added nanocomposite solution and stirred (400 rpm) for 24 h at room temperature. rGO-MNP-PDA nanocomposite was centrifuged at 14.000 rcf (g), washed with distilled water and ethanol three times, and left to dry at 60 °C [17].

The rGO-PDA and MNP-PDA were prepared based on the ratios of preparing the rGO-MNP-PDA. 200 mg GO was added into Tris buffer (0.01 M, pH 8.5), and sonicated (ultrasonic bath (ultrasonic power: 200 W, ultrasonic frequency: 37 kHz) for 30 min. Then, 400 mg DA was added nanocomposite solution and stirred (400 rpm) for 24 h at room temperature. Then, rGO-PDA nanocomposite was centrifuged at 14.000 rcf (g), washed with distilled water and ethanol three times, and left to dry at 60 °C. Likewise, 200 mg MNP was added into Tris buffer (0.01 M, pH 8.5), and sonicated (ultrasonic bath (ultrasonic power: 200 W, ultrasonic frequency: 37 kHz) for 30 min. Then, 400 mg DA was added to the nanocomposite solution and stirred (400 rpm) for 24 h at room temperature. Then, MNP-PDA nanocomposite was centrifuged at 14.000 rcf (g), washed with distilled water and ethanol three times, and left to dry at 60 °C.

### 2.5. Preparation of PS/rGO-MNP-PDA, PS/rGO-PDA and PS/MNP-PDA Nanofibers

PS/rGO-MNP-PDA, PS/rGO-PDA and PS/MNP-PDA nanofibers were obtained by adding 0.5% (w/v) rGO-MNP-PDA, rGO-PDA, and MNP-PDA nanocomposite to 35% (w/v) PS solution in DMF, then stirred (300 rpm) at 50 °C overnight. Then 2.0 mL of all nanocomposite solutions were taken into a syringe (metal needle diameter of 8.8 mm). Nanocomposite based nanofibers were deposited on the working electrode (4 mm) of SPCE by blocking from adhering to the surface of the reference and counter electrodes during the electrospinning process. Electrospun nanofibers were produced by electrospinning technique under the defined optimum conditions (tip-to-collector distance (TCD): 16 cm, applied voltage: 7.6 kV, flow rate: 1.2 mL/h, temperature: 26 °C, humidity: 57%). All nanofibers modified SPCEs were dried at room temperature overnight prior to performing immobilization of Anti-CRP. 

### 2.6. Preparation of PS/rGO-MNP-PDA/Anti-CRP, PS/rGO-PDA/Anti-CRP, and PS/MNP-PDA/Anti-CRP

Anti-CRP immobilization was carried out directly on the PS/rGO-MNP-PDA, PS/rGO-PDA, and PS/MNP-PDA nanofibers modified SPCE. Stock Anti-CRP solution was diluted 1000 times in PBS (0.01 M, pH 7.4) [34]. Then, 5.0 µL of antibody solution was added onto PS/rGO-MNP-PDA, PS/rGO-PDA and PS/MNP-PDA nanofibers modified SPCEs and incubation was carried out for 2 h at room temperature. All electrodes were washed to remove unbound Anti-CRP with distilled water before electrochemical measurements. 

### 2.7. Electrochemical Measurements

**Scheme 1:**
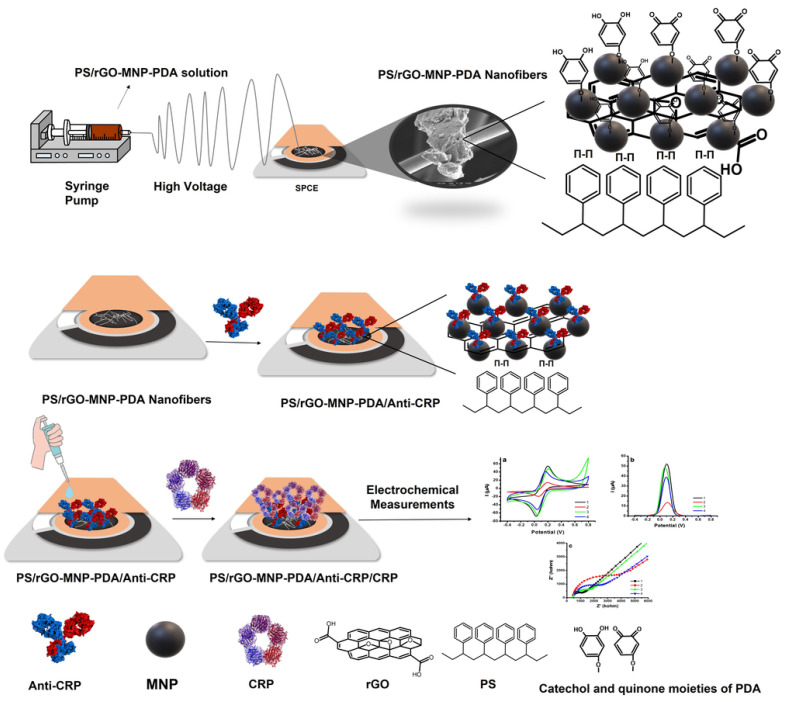
Schematic representation of the preparation of PS/rGO-MNP-PDA/Anti-CRP modified SPCE and electrochemical detection of CRP.

A PalmSens4 instrument coupled to a computer (PSTrace 5.8 software) was used to perform CV, DPV, EIS measurements for bare SPCE, PS/rGO-MNP-PDA nanofiber modified SPCE, and PS/rGO-MNP-PDA/Anti-CRP modified SPCE. Measurements of CV and DPV were carried out at a scan rate of 50 mV/s between −0.4 to +0.8 V. EIS trial was performed in the excitation voltage at 0.18 V, frequency range of 0.21 × 10^−4^–100 kHz, and DC potential at 10 mV. All measurements were carried out in 10 mM phosphate buffer saline (PBS, pH 7.4) including 5.0 mM hexacyanoferrate(III) (HCF, redox probe) and 0.1 M KCl. A schematic representation of PS/rGO-MNP-PDA/Anti-CRP modified SPCE and electrochemical detection of CRP is demonstrated in Scheme 1.

## 3. Results and Discussion

### 3.1. Characterization of GO and rGO-MNP

**Figure 1:**
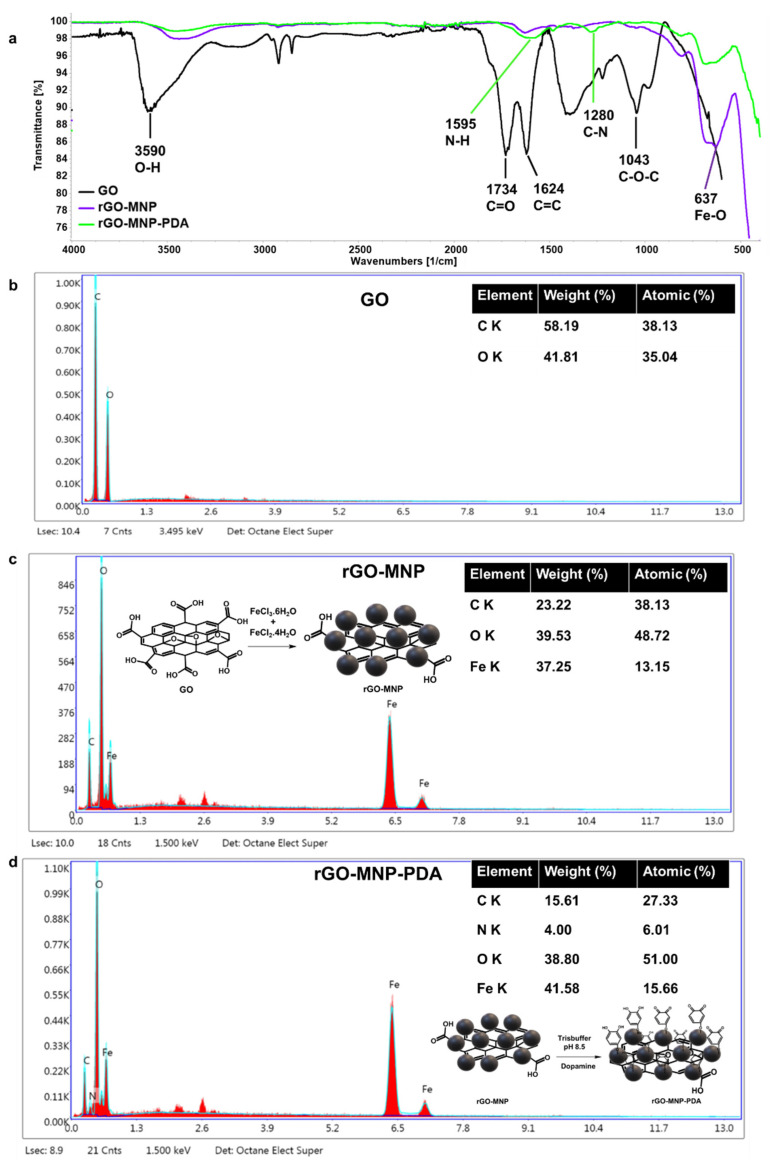
(**a**) FTIR spectrum of GO, rGO-MNP, rGO-MNP-PDA, (**b**) EDS spectrum of GO, (**c**) EDS spectrum of rGO-MNP, and (**d**) EDS spectrum of rGO-MNP-PDA.

Characterization of the obtained GO, MNP, rGO-MNP, and rGO-MNP-PDA was obtained by FTIR. Figure 1a shows the FTIR spectrum of GO, MNP, rGO-MNP, and rGO-MNP-PDA. After oxidation process of GO, the stretching vibration of C=O (1734 cm^−1^), epoxy stretching of C-O-C (1043 cm^−1^), and stretching vibration of H-bonded of -OH (3590 cm^−1^) functional groups were observed in the FTIR spectrum of GO [16,35]. According to the FTIR spectrum of rGO-MNP, it was observed that the functional groups of GO disappeared due to the formation of the Fe-O-C bond during formation of the rGO-MNP nanocomposite [36]. The Fe-O specific band of MNP appeared at 637 cm^−1^ [37]. Hydroxylated metal or metal oxide surfaces may chelate with catechol groups of polydopamine [38]. In FTIR spectrum of rGO-MNP-PDA, the presence of amine groups emerged with the formation of polydopamine on rGO-MNP nanosheets, as observed at 1595 cm^−1^ (N-H) and 1280 cm^−1^ (C-N) [17,39].

EDS analysis was also performed to investigate the further characterization (elemental percentages of iron (Fe), nitrogen (N), and oxygen (O)) of rGO-MNP-PDA nanocomposite for each modification step. According to the EDS spectra of GO, the presence of an oxygen peak (35.04%, atomic) is evidence that GO synthesis was carried out successfully (Figure 1b). After the modification of GO with MNP, an iron peak (13.15%, atomic) was observed in the EDS spectrum of rGO-MNP. It is thought that the increased oxygen ratio was another proof of successful modification with MNP (Fe_3_O_4_) (Figure 1c). Then, a nitrogen peak (6.01%, atomic) was observed owing to PDA formation on the rGO-MNP nanosheets (Figure 1d). EDS and FTIR results were found as compatible with each other for each modification step.

**Figure 2:**
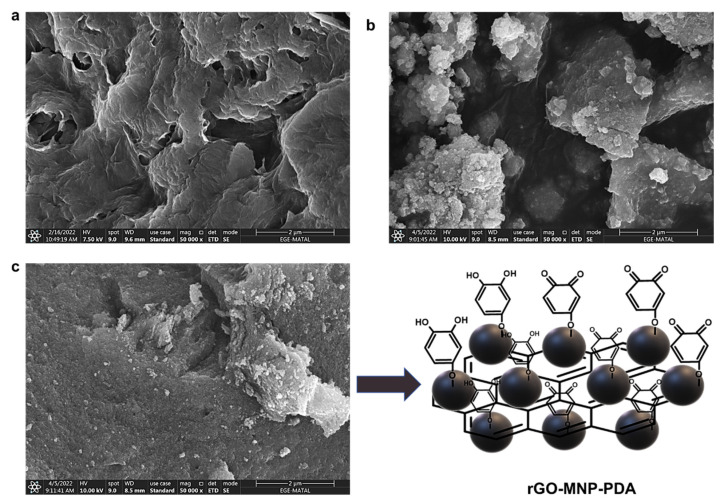
SEM images (50.000× magnification) of (**a**) GO, (**b**) rGO-MNP, and (**c**) rGO-MNP-PDA.

SEM images were taken to examine how to change the surface morphology of rGO-MNP-PDA nanocomposite after each modification step. A SEM image of non-modified GO nanosheets is shown in Figure 2a. MNPs tend to aggregate due to their hydrophobic nature. Aggregated MNPs were observed on the GO nanosheets (Figure 2b) after the modification of GO with MNP. The surface of the rGO-MNP nanocomposite appeared to become smoother after coating with PDA (Figure 2c).

### 3.2. Characterization of PS/rGO-MNP-PDA Electrospun Nanofibers

Each parameter (applied voltage, polymer concentration, solvent properties, TCD, flow rate, temperature, and humidity) must be optimized in the electrospinning process to obtain the best morphological properties [40]. In the literature studies, it was indicated that DMF is the most suitable solvent to produce smooth and bead-free PS nanofibers due to its high dielectric constant (36.4) [41,42,43]. In addition, we optimized all electrospinning parameters to produce PS nanofibers with optimum morphological properties in our previous study [44]. Hence, DMF was chosen as a solvent for the PS (35%; w/v). 

**Figure 3:**
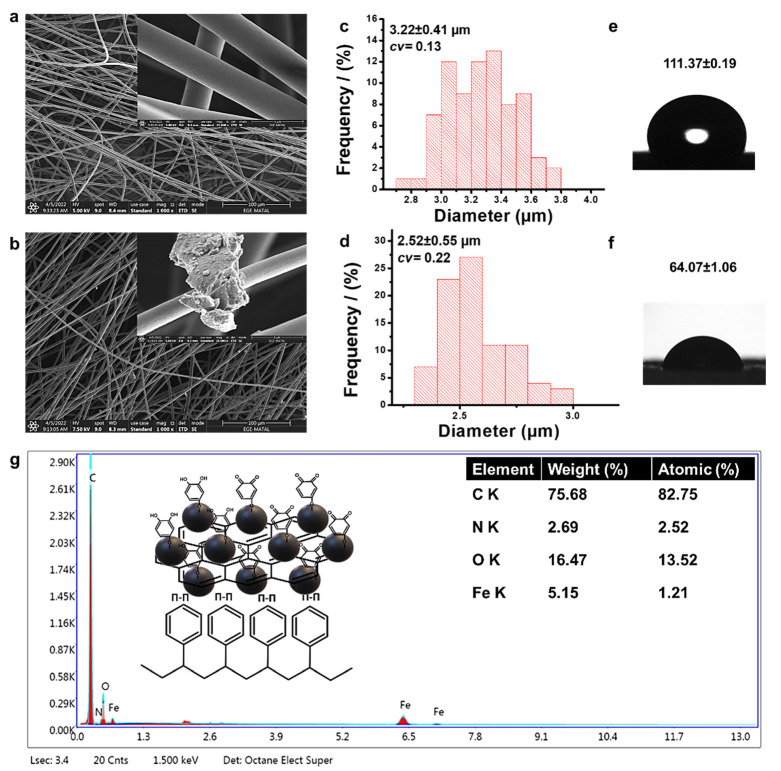
SEM images (1000× magnification) of (**a**) PS (35%) (w/v), (**b**) PS/rGO-MNP-PDA 0.5% (w/v), (interior SEM images (25.000× magnifications); diameter distribution graphics of nanofibers (**c**) PS (35%) (w/v), (**d**) PS/rGO-MNP-PDA 0.5% (w/v) electrospun nanofibers, contact angle measurements of (**e**) PS (35%) (w/v), (**f**) PS/rGO-MNP-PDA 0.5% (w/v) electrospun nanofibers, and (**g**) EDS spectrum of PS/rGO-MNP-PDA 0.5% (w/v) electrospun nanofibers (electrospinning process conditions: 7.6 kV (applied voltage), 16 cm (TCD), and 1.2 mL/h (solution flow rate).

PS and PS/rGO-MNP-PDA nanofibers were produced under the determined optimum electrospinning conditions and their morphological properties were examined with the SEM. 0.5% (w/v) rGO-MNP-PDA nanocomposite was added to the 35% (w/v) PS polymer solution in the DMF to produce PS/rGO-MNP-PDA nanofibers. According to SEM images of PS and PS/rGO-MNP-PDA nanofibers, we could obtain smooth and bead-free nanofibers (Figure 3a,b). Notably, the formation of rGO-MNP-PDA nanocomposite on the PS nanofibers can be seen clearly in Figure 3b. This is not only the main difference between both SEM images but proof of producing PS/rGO-MNP-PDA nanofibers successfully. Nanofiber diameter distributions were calculated by marking 70 different points of nanofibers in each SEM image via the Image J program. According to the results, it was found that the diameter distributions of PS and PS/rGO-MNP-PDA nanofibers were 3.22 ± 0.41 and 2.52 ± 0.55 µm, respectively (Figure 3c,d). It was observed that decreased nanofiber diameters were obtained with the integration of rGO-MNP-PDA nanocomposite to PS polymer solution. This phenomenon can be ascribed to the presence of Fe_3_O_4_ in the nanocomposite. Fe_3_O_4_ MNPs, based on a semi-metallic metal oxide with an inverted spinel structure, have high electrocatalytic properties, magnetic, and natural electrical conductivity [25]. Since conductivity has an important role in nanofiber diameter and Taylor cone formation, it is the key factor in the electrospinning process. The Taylor cone is the most important formation process in the electrospinning process. This phenomenon is controlled with the electrostatic force created on the surface of the needle tip drop of the polymer solution together with the external electric field. The increased conductivity of the polymer solution leads to an increase in the charge of the polymer solution to create the Taylor cone and leads to decreased nanofiber diameter due to offering enough free charges on the surface of the polymer solution [40,45,46]. Hence, we obtained decreased nanofiber diameters with the integration of rGO-MNP-PDA nanocomposite to PS polymer solution. Additionally, the coefficient of variation (cv) values of nanofiber diameters were calculated as 0.13 and 0.22 for PS and PS/rGO-MNP-PDA nanofibers, respectively. The fact that the calculated cv values are less than 0.3 indicates that the obtained nanofibers are uniform [47]. The success of the immobilization process is highly related to the wettable properties of the immobilization matrix for biological molecules [48]. Since PS has a hydrophobic character [30], we aimed to obtain a hydrophilic immobilization matrix for Anti-CRP and produce highly conductive nanofibers while combining PS and rGO-MNP-PDA nanocomposite. The contact angle of PS and PS/rGO-MNP-PDA nanofibers were found as 111.37 ± 0.19 and 64.07 ± 1.06, respectively (Figure 3e,f). Decreased contact angle value was observed with the integration of rGO-MNP-PDA nanocomposite to PS polymer solution due to the presence of PDA on the PS nanofiber surface. EDS analysis was carried out to examine the elemental composition of PS/rGO-MNP-PDA nanofibers (Figure 3g). According to the EDS results, elemental composition percentages of carbon (C), N, O, and Fe were found to be 82.75%, 2.52%, 13.52%, 1.21%, respectively. The presence of N and Fe in the EDS spectrum indicated that the production of PS/rGO-MNP-PDA nanofibers was carried out successfully.

### 3.3. Electrochemical Characterization of PS/rGO-MNP-PDA/Anti-CRP

**Figure 4:**
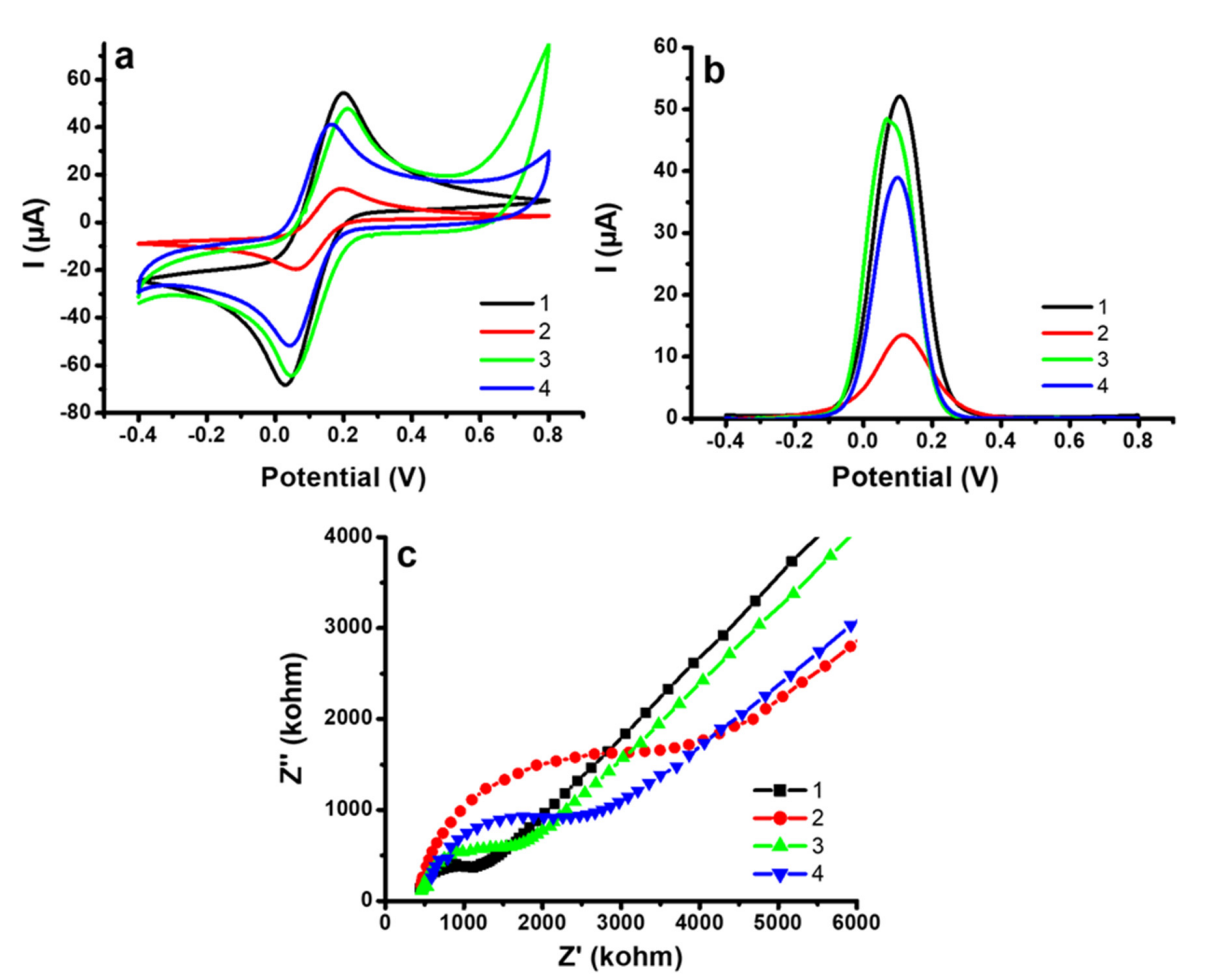
(**a**) CV profile, bare SPCE (1) PS/rGO-MNP-PDA (2) PS/rGO-MNP-PDA/Anti-CRP (3), and PS/rGO-MNP-PDA/Anti-CRP/CRP (4) (**b**) DPV profile, bare SPCE (1) PS/rGO-MNP-PDA (2) PS/rGO-MNP-PDA/Anti-CRP (3), and PS/rGO-MNP-PDA/Anti-CRP/CRP (4), (**c**) Nyquist profile, bare SPCE (1) PS/rGO-MNP-PDA (2) PS/rGO-MNP-PDA/Anti-CRP (3), and PS/rGO-MNP-PDA/Anti-CRP/CRP (4), (All measurements were carried out in 10 mL PBS (pH 7.4) including 0.1 M KCl and 5.0 mM HCF, [CRP] = 20 ng/mL).

Electrochemical characterizations of bare, PS/rGO-MNP-PDA, PS/rGO-MNP-PDA/Anti-CRP, and PS/rGO-MNP-PDA/Anti-CR/CRP modified SPCE surfaces were performed by CV, DPV, and EIS measurements. Cyclic voltammetry (CV) is one of the popular voltametric techniques that provides information about the qualitative aspects of electrochemical reactions [49]. According to CV voltammograms, oxidation and reduction peaks of anionic redox solution (hexacyanoferrate (III), HCF) were detected in all of the CV profiles and I_anodic_ values were found to be 68.67 µA, 16.62 µA, 52.80 µA, 45.32 µA for bare SPCE, PS/rGO-MNP-PDA, PS/rGO-MNP-PDA/Anti-CRP, PS/rGO-MNP-PDA/Anti-CRP/CRP modified SPCEs, respectively (Figure 4a). A decreased current was obtained after the deposition of PS/rGO-MNP-PDA nanofibers on bare SPCE. This phenomenon can be attributed to the entrapment of the redox-active species in the HCF solution into the porous PS/rGO-MNP-PDA nanofibers. Hence, a redox reaction could not occur easily on the electrode surface. Additionally, this is an indication that the system exhibits thin layer diffusion [50]. After the conjugation of Anti-CRP on the PS/rGO-MNP-PDA nanofibers modified SPCE, an increased current was observed in the CV profile. Antigen-binding regions (F_ab_)_2_ of antibodies are usually positively charged in PBS (pH 7.4), and hence electrostatic attraction occurred between HCF and PS/rGO-MNP-PDA/Anti-CRP modified SPCE [51,52]. Consequently, the increased electrochemical current was observed with the increased electron transfer rate [53,54]. The obtained CV profile was very similar to the observations of Ozoemena and co-workers. They developed an electrochemical immunosensor with fiber hybrids for the detection of *Vibrio cholerae* toxin. After the modification of the working electrode with fiber, a decreased current was observed in the CV profile. They attributed this occurrence to the electrode surface porosity. After the conjugation of the antibody on the fiber-modified electrode surface, they observed an increased current compared to the fiber-modified electrode [55]. Consequently, it was found that the obtained CV profile in our study is compatible with the literature.

DPV is utilized for quantitative analysis as well as to investigate the thermodynamics of chemical reactions due to its low background properties and reduced capacitive current. Although the scanning speed is relatively slow, its selectivity is higher than other methods. The current is measured before and after the pulse is applied and the difference is taken to reduce the share of capacitive current in the measured current and increase the selectivity. DPV can make peak discrimination even in small wave ranges (0.04 to 0.05 V); hence, this technique was chosen for quantitative CRP detection in this study [56,57]. Figure 4b shows the DPV profiles of bare SPCE, PS/rGO-MNP-PDA, PS/rGO-MNP-PDA/Anti-CRP, PS/rGO-MNP-PDA/Anti-CRP/CRP modified SPCEs, respectively. As in the CV curve, decreased current was obtained when the electrode surface was covered with PS/rGO-MNP-PDA nanofiber, and an increase was observed after the immobilization of Anti-CRP on the PS/rGO-MNP-PDA nanofiber modified SPCE. Thus, DPV profiles of all modification steps were found to be compatible with the CV profile.

EIS is a valuable electrochemical technique that offers us information about the reactions occurring at the interface of the electrode and the electron transfer resistance. Notably, the semi-circle of the Nyquist plot (faradaic impedance spectrum), which is inversely proportional to the electrical conductivity, offers us information on how the charge transfer resistance changes with each modification step. The immobilization of the antibody or the occurrence of antibody-antigen immunoreaction on the electrode surface prohibits the spreading of redox-active species on the electrode surface due to creating an insulating layer. This event creates a change in capacitance, causing a change in the EIS profile of the electrodes [58,59]. The increased ion charge transfer resistance (R_ct_) was observed after the modification of the bare SPCE with PS/rGO-MNP-PDA nanofiber in the Nyquist profile. Then, decreased R_ct_ was obtained after the immobilization of Anti-CRP with PS/rGO-MNP-PDA nanofiber. Finally, increased R_ct_ was observed again with the occurrence of immunoreaction of CRP with Anti-CRP on the modified electrode surface (Figure 4c). Consequently, all results indicated that all electrochemical profiles were compatible with each other. 

### 3.4. Application of PS/rGO-MNP-PDA/Anti-CRP for CRP Detection

**Figure 5:**
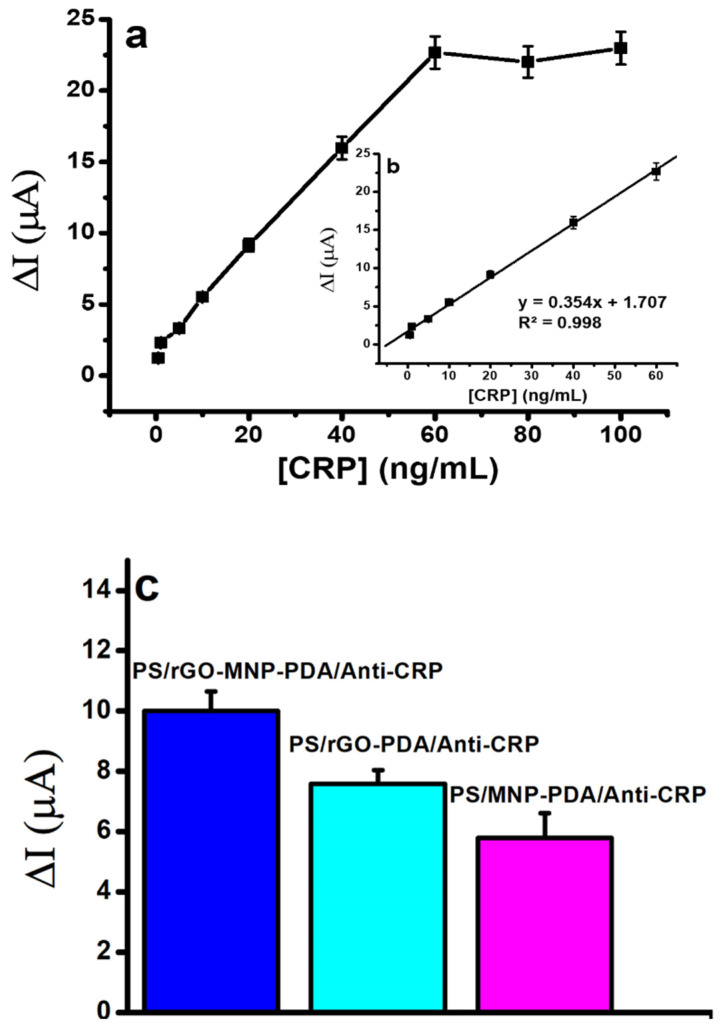
(**a**) Impact of CRP concentration on the immunosensor current response, (**b**) Linear detection range of CRP, (**c**) CRP detection with PS/rGO-MNP-PDA/Anti-CRP, PS/rGO-PDA/Anti-CRP, and PS/MNP-PDA/Anti-CRP immunosensors. (All measurements, scan rate: 50 mVs^−1^, in the presence of 10 mL PBS (pH 7.4) solution including 5 mM HCF and 0.1 M KCl, [CRP] = 20 ng/mL. Error bars show the standard deviation of at least 3 replicate measurements).

DPV measurements were performed at different concentrations of CRP (0.5–100 ng/mL) to determine the linear detection range of CRP via using the PS/rGO-MNP-PDA/Anti-CRP immunosensor. The linear detection range of CRP was determined as 0.5–60 ng/mL. The equation of the calibration curve was determined as y = 0.354x + 1.707 (R^2^ = 0.998). There was no significant change in intensity of current after 60 ng/mL CRP concentration owing to steric hindrance created by CRP. CRP concentrations in the saliva of healthy adults range from 0.05 to 64.30 ng/mL [60]. Thus, the developed PS/rGO-MNP-PDA/Anti-CRP immunosensor platform was found to be suitable to determine the amount of CRP in the saliva. Figure 5 shows the influence of CRP concentration on the immunosensor current response and the linear detection range for CRP.

LOD is described as the lowest concentration of analyte that can be determined at a certain level of confidence [61]. A total of 10 different immunosensors were prepared and DPV measurements were carried out with 0.5 ng/mL CRP concentration, which is the lowest point of the calibration curve for the detection the LOD of the PS/rGO-MNP-PDA/Anti-CRP immunosensor. LOD was determined using the 3SD/m formulation (SD: standard deviation of 10 measurements values performed at 0.5 ng/mL CRP concentration, m: the slope of the CRP calibration curve) [62,63,64,65]. Consequently, the LOD was calculated as 0.33 ng/mL for CRP.

PS/rGO-PDA/Anti-CRP and PS/MNP-PDA/Anti-CRP immunosensors were prepared and the effect of each component in the nanocomposite-based electrospun nanofiber (MNP, rGO) on the sensor response was investigated using the DPV technique in the presence of 20 ng/mL CRP (Figure 5). The rGO-PDA and MNP-PDA nanocomposites were prepared based on the ratios of preparing the rGO-MNP-PDA and their nanofibers were produced under the determined optimum conditions with PS/rGO-MNP-PDA nanofibers. According to the results, while the PS/rGO-PDA/Anti-CRP immunosensor exhibited sensor response (7.591 ± 0.45) close to the PS/rGO-MNP-PDA/Anti-CRP immunosensor (10.010 ± 0.64), the PS/MNP-PDA/Anti-CRP immunosensor exhibited a lower performance (5.798 ± 0.82) than both. Teymourian et al. determined the effective surface areas of magnetic nanoparticles/reduced graphene oxide nanosheets modified glassy carbon (Fe_3_O_4_/r-GO/GC), rGO/GC, and Fe_3_O_4_/GC electrodes. They found the effective surface areas to be Fe_3_O_4_/r-GO/GC (0.0442 cm^−2^) > r-GO/GC (0.0428 cm^−2^) > Fe_3_O_4_/GC (0.0308 cm^−2^), respectively [66]. These results show that increased effective specific surface area can be obtained by integrating MNPs into the graphene structure. Thus, immunosensors with improved performance can be produced. It is thought that PS/rGO-MNP-PDA nanofibers modified SPCE exhibited increased effective specific surface area compared to those modified with rGO-PDA or MNP-PDA alone. Depending on the increased effective specific surface area, more PDA surface was covered, and a higher amount of Anti-CRP was bound on the electrode surface. Thus, more CRP protein binding to the surface of PS/rGO-MNP-PDA/Anti-CRP immunosensor was obtained than with other immunosensors. Accordingly, it can be said that PS/rGO-MNP-PDA nanofibers obtained by combining rGO and MNP create synergistic effects and provide more conduction paths for electron transfer to the anionic redox probe (HCF). Additionally, the low CRP binding to the PS/MNP-PDA/Anti-CRP immunosensor can be attributed to the low effective specific surface area due to the aggregation tendency of MNPs. Moreover, the linear detection range and LOD were determined for these immunosensors platforms separately. Linear detection ranges of PS/rGO-PDA/Anti-CRP and PS/MNP-PDA/Anti-CRP immunosensors were determined as 1–40 ng/mL and 2–40 ng/mL CRP, respectively. LOD values of PS/rGO-PDA/Anti-CRP and PS/MNP-PDA/Anti-CRP immunosensors were calculated as 0.89 ng/mL and 1.64 ng/mL for CRP, respectively.

Precision is an illustration of how close the independent measurement results are to each other. Repeatability is the precision achieved under repeatability conditions [67]. SD and cv% are used to indicate the precision of a set of repeated data. Low cv% and SD values have remarkable importance for immunosensors as they reveal the reliability and repeatability of the developed system. The repeatability studies were performed with 10 prepared immunosensors separately, and DPV measurements were carried out in the presence of 20 ng/mL CRP. SD and cv% values were determined as ± 0.22 and 2.31%, respectively. A cv% value below 5% increases the precision of the method [57,68]. It can be said that the PS/rGO-MNP-PDA/Anti-CRP immunosensor provides us high precision.

**Figure 6:**
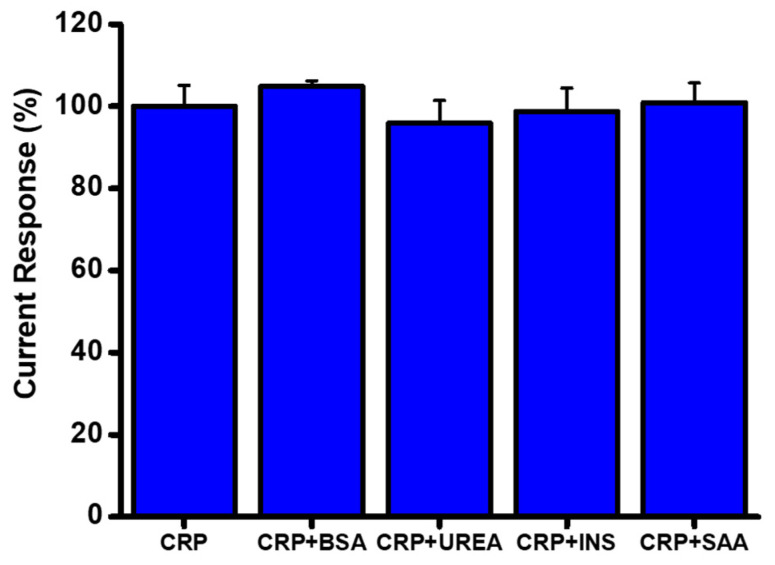
Interference effect of BSA, urea, INS, and SAA on the detection of CRP using PS/rGO-MNP-PDA/Anti-CRP (in the presence of 10 mL PBS (pH 7.4) solution containing 5 mM HCF and 0.1 M KCl; [CRP]: 20 ng/mL, [BSA]; 1 mg/mL, [Urea]:0.5 mg/mL, [INS]: 1 µU/mL, [SAA]: 20 µg/mL; scan rate: 50 mVs^−1^.) Error bars show the standard deviation of at least 3 replicate measurements.

The interference effects of BSA, urea, INS and SAA, which are some biological substances in saliva that may interfere with the PS/rGO-MNP-PDA/Anti-CRP immunosensor response developed for CRP detection, were investigated. The reference ranges of BSA (0.72–2.45 mg/mL) and urea (39–66 mg/dL) in saliva were determined according to the literature [69,70]. It was reported in the literature that SAA concentrations are generally between 20 and 50 mg/L for humans [71]. INS should be lower than 12 µU/mL in blood for healthy persons [44,72]. However, the INS level in saliva is 10 times lower than its blood concentration [73]. The selectivity of the developed PS/rGO-MNP-PDA/Anti-CRP immunosensor was examined in the presence of 20 ng/mL CRP solutions containing 1 mg/mL BSA, 0.5 mg/mL urea, 1 µU/mL INS, and 20 µg/mL SAA through DPV measurements. It was determined that the selectivity was 104.86% for CRP+BSA, 95.9% for CRP+Urea, 98.76% for CRP+INS, and 100.91% for CRP+SAA (Figure 6). It can be said that BSA, urea, INS and SAA together with CRP did not have any interference effect on the developed PS/rGO-MNP-PDA/Anti-CRP immunosensor response.

### 3.5. Sample Application

Recovery studies provide information on the accuracy of the system. For this reason, nine PS/rGO-MNP-PDA/Anti-CRP immunosensors were prepared separately. The recovery % was determined using a known amount of CRP (20 ng/mL) added to artificial saliva and concentration of CRP measured with PS/rGO-MNP-PDA/Anti-CRP. The recovery % performed with artificial saliva is shown in Table 1. The recovery % of CRP detection was calculated as 100.36%. Considering that the acceptable recovery percentages are 95–105%, this result confirms the validity of the system [74,75,76].

Biosensors developed for CRP detection in the literature are given in Table 2 for comparison with the results in our study. It can be seen that the PS/rGO-MNP-PDA/Anti-CRP has better detection limits for the detection of CRP compared to the literature.

## 4. Conclusions

In this study, besides the development of a new immunosensor for non-invasive detection of CRP from artificial human saliva, the effect of each nanomaterial on the sensor response in the PS/rGO-MNP-PDA nanofibers was also investigated. The PS/rGO-MNP-PDA/Anti-CRP showed a good linearity with CRP concentration in the range of 0.5–60 ng/mL, and the LOD was 0.33 ng/mL. Additionally, PS/rGO-PDA/Anti-CRP and PS/MNP-PDA/Anti-CRP immunosensors were prepared and the linear detection range and LOD were determined for these immunosensors platforms under the electrochemical measurement. It was determined that the PS/rGO-MNP-PDA/Anti-CRP immunosensor exhibited better performance than the others as a result of the increased effective specific surface area and the synergistic effect between the MNPs and rGO. The only limitation of the developed immunosensor is that it is disposable. Additionally, the use of biological molecules increases the cost. However, considering the cost of ELISA kits today, the cost of the developed immunosensor is quite low. It is thought that the reported PS/rGO-MNP-PDA/Anti-CRP immunosensor has the potential for rapid diagnosis of CRP from human saliva with high sensitivity in the clinical field. Moreover, the PS/rGO-MNP-PDA nanofibers can be a good immobilization platform for other biomolecules.

## Figures and Tables

**Table 1:** Recovery% of CRP in artificial saliva with PS/rGO-MNP-PDA/Anti-CRP immunosensor (n = 9).

Artificial Sample	Added CRP (ng/mL)	Found CRP (ng/mL)	cv %	Recovery %
Saliva	20.00	20.07 ± 0.62	3.09	100.35

**Table 2:** Some biosensor studies developed for CRP detection.

Analyte	Technique	Linear Range (ng/mL^−1^)	LOD (ng/mL^−1^)	References
**CRP**	LFA	1–10^4^	55	[77]
**CRP**	LFIA	10–100	1	[78]
**CRP**	PIS	5–100	5	[79]
**CRP**	PTB	0.1–100	0.1	[80]
**CRP**	EIS	10–10^4^	19.38	[81]
**CRP**	**DPV**	**0.5–60**	**0.33**	**This study**

LFIA: Lateral flow immunoassay; LFA: Lateral flow assay, PTB: Photothermal biosensor, PIS: plasmonic–photonic imaging sensor.

## Data Availability

Not applicable.
